# Low risk of intra-abdominal infections in rectal cancer patients treated with Hartmann’s procedure: a report from a national registry

**DOI:** 10.1007/s00384-018-2967-0

**Published:** 2018-01-21

**Authors:** Ingvar Sverrisson, Maziar Nikberg, Abbas Chabok, Kenneth Smedh

**Affiliations:** Colorectal Unit, Department of Surgery and Centre for Clinical Research of Uppsala University, Västmanland’s Hospital Västerås, SE -72189 Västerås, Sweden

**Keywords:** Rectal cancer, Surgery, Hartmann’s procedure, Postoperative complications, Intra-abdominal infections

## Abstract

**Purpose:**

To describe the postoperative surgical complications in patients with rectal cancer undergoing Hartmann’s procedure (HP).

**Methods:**

Data were retrieved from the Swedish Colorectal Cancer Registry for all patients with rectal cancer undergoing HP in 2007–2014. A retrospective analysis was performed using prospectively recorded data. Characteristics of patients and risk factors for intra-abdominal infection and re-laparotomy were analysed.

**Results:**

Of 10,940 patients resected for rectal cancer, 1452 (13%) underwent HP (median age, 77 years). The American Society of Anesthesiologists (ASA) score was 3–4 in 43% of patients; 15% had distant metastases and 62% underwent a low HP. The intra-abdominal infection rate was 8% and re-laparotomy rate was 10%. Multivariable logistic regression analysis identified preoperative radiotherapy (OR, 1.78; 95% CI, 1.14–2.77), intra-operative bowel perforation (OR, 1.99; 95% CI, 1.08–3.67), T4 tumours (OR, 1.68; 95% CI 1.04–2.69) and female gender (OR, 1.73; 95% CI, 1.15–2.61) as risk factors for intra-abdominal infection. ASA score 3–4 (OR, 1.62; 95% CI, 1.12–2.34), elevated BMI (OR, 1.05; 95% CI, 1.02–1.09) and female gender (OR, 2.06; CI, 1.41–3.00) were risk factors for re-laparotomy after HP. The rate of intra-abdominal infection was not increased after a low HP.

**Conclusions:**

Despite older age and co-morbidities including more advanced cancer, patients undergoing Hartmann’s procedure had low rates of serious postoperative complications and re-laparotomy. A low HP was not associated with a higher rate of intra-abdominal infection. HP seems to be appropriate for old and frail patients with rectal cancer.

## Introduction

Survival and local recurrence rates after rectal cancer have improved since the introduction of total mesorectal excision and preoperative radiotherapy (RT) [1, 2]. The number of patients with rectal cancer treated surgically with Hartmann’s procedure (HP) is increasing, especially among old and frail patients with metastatic disease [3].

Postoperative complications have been reported after HP performed to treat diverticular disease and sigmoid cancer with transection of the proximal rectum [4, 5]. However, the morbidity after HP in patients with rectal cancer and distal transection of the rectum has been rarely reported. Some reports have shown a high frequency of postoperative complications such as pelvic abscesses—especially after low HP—and increased rates of re-operation and re-admission [6–8]. Abdomino-perineal excision (APE) is performed in some centres to reduce the risk of pelvic abscesses [7]. However, most previous studies have been small, retrospective and not population based, and do not provide clear conclusions about which procedure is best suited for old and frail patients.

The aim of this study was to examine the postoperative complication pattern in rectal cancer patients undergoing HP using data from the population-based Swedish Colorectal Cancer Registry (SCRCR).

## Methods

All hospitals in Sweden (about 10 million inhabitants) that treat patients with colorectal cancer report clinical, surgical, pathological and follow-up data to the SCRCR, which has a coverage of more than 99% of all patients diagnosed with rectal cancer. A detailed description of the registry, which was originally launched as the Swedish Rectal Cancer Registry in 1995, has been reported [9], as has validation of postoperative complications in the registry database [10, 11]. In the SCRCR, a rectal cancer is defined as an adenocarcinoma of the rectum within 15 cm of the anal verge.

According to the Swedish national guidelines, transection of the rectum in patients requiring partial mesorectum excision is recommended 5 cm distal to the tumour. Patients with a tumour within 10 cm from the anus and registered as having undergone HP were regarded to have had a low HP, which was defined as transection of the rectum below the peritoneal reflection just above the levator ani. A local radical resection was defined as curative by the surgeon and radical by the pathologist (R0). Between 2007 and 2014, 10,940 patients aged 18 years and older underwent bowel resection for rectal cancer. Emergency HPs were excluded. In most patients, the decision was made to perform a permanent colostomy without later reconstruction. Postoperative complications were defined as occurring within 30 days of the primary operation.

During the study period, the most common preoperative RT regime was a short course (5 Gy five times in 1 week) followed immediately by surgery. For patients receiving preoperative chemoradiotherapy (CRT), a long course of RT (1.8–2 Gy × 25–28 over 5–6 weeks) was delivered together with chemotherapy, usually with capecitabine.

### Statistical analysis

Data were analysed using SPSS IBM Statistics (v. 22; IBM Corp., Armonk, NY, USA). Differences in proportions were calculated using the chi-square test or the *t* test for independent samples as appropriate. A *p*-value of <0.05 was considered significant. Binary univariable logistic regression analyses to identify the independent risk factors for intra-abdominal infection and re-laparotomy within 30 days postoperatively were performed. The variables with a *p*-value <0.05 in the univariable analysis were analysed in a multivariable model, and those that remained significant are presented. Data are reported as the odds ratio (OR) with 95% confidence interval (CI).

## Results

Of 10,940 patients resected for rectal cancer, 1452 (13%) underwent HP. In 62% of the patients, the distance of the tumour from the anal verge was 10 cm or less, which means that the rectum was transected just above the levator ani and corresponded to a low HP. The median age was 77 years, and 43% had an American Society of Anesthesiologists (ASA) score of 3 or 4 (Table 1). An accredited colorectal surgeon or a surgeon with a special interest in colorectal surgery, trained in the total mesorectum excision technique, performed or supervised 97% of the operations. Fifty per cent (*n* = 720) of the patients received preoperative radiotherapy, and 201 of these received concomitant chemotherapy. Intra-operative bowel perforation occurred in 8% of patients. The overall and surgical complication rates were 41% and 26%, respectively (Table 2). Intra-abdominal infections occurred in 8% of the patients, and the re-laparotomy rate was 10%. The 30- and 90-day mortality rates were 4% and 6%, respectively.

**Table 1:** Clinical characteristics of all patients with rectal cancer treated with Hartmann’s procedure in Sweden between 2007 and 2014

	HP *n* = 1452
Age^a^ in years	77 (19–98)
Gender	
Male	761 (52)
Female	691 (48)
BMI^a^	25.0 (13.7–48.0)
ASA score 3/4	628 (43)
Staging (MRI)	
T1–2	282 (19)
T3	692 (48)
T4	247 (17)
N1/2	563 (39)
Distant metastases	217 (15)
Distance of tumour from anal verge (cm)	
0–5	113 (8)
6–10	783 (54)
11–15	527 (36)
Missing	29 (2)
Preoperative RT	720 (50)
Laparoscopy	71 (5)
Rectal wash-out	980 (68)
Intra-operative bowel perforation	115 (8)
Local radical resection	1298 (89)

Values in parentheses are percentages unless otherwise indicated

^a^Values represent median (range)

HP Hartmann’s procedure, BMI body mass index, ASA American Society of Anesthesiologists, MRI magnetic resonance imaging, RT radiotherapy

**Table 2:** Postoperative complications after Hartmann’s procedure in patients with rectal cancer treated in Sweden between 2007 and 2014

	HP *n* = 1452
Overall complications	594 (41)
Overall surgical complications	372 (26)
Intra-abdominal infections	122 (8)
Unplanned ICU stay	138 (10)
Re-laparotomy	146 (10)
30-day mortality	53 (4)
90-day mortality	87 (6)

Values in parentheses are percentages. HP Hartmann’s procedure, ICU intensive care unit

The multivariable logistic regression analysis of patients treated with HP identified preoperative radiotherapy (OR 1.78; 95% CI, 1.14–2.78), intra-operative bowel perforation (OR 1.99; 95% CI, 1.08–3.67), T4 tumours (OR 1.68; 95% CI 1.04–2.69) and female gender (OR 1.73; 95% CI, 1.15–2.61) as risk factors for intra-abdominal infection (Table 3). Elevated body mass index (BMI) (OR 1.05; 95% CI 1.02–1.09), female gender (OR 2.06; 95% CI 1.41–3.00) and higher ASA score (OR 1.62; 95% CI 1.12–2.34) were associated with an increased risk of re-laparotomy (Table 4). There were no differences between men and women in terms of age (*p* = 0.064), BMI (*p* = 0.613), T4 tumours (*p* = 0.237), metastases (*p* = 0.429) or ASA score (*p* = 0.398). A low HP was not significantly associated with a higher rate of intra-abdominal infection.

**Table 3:** Binary univariable and multivariable logistic regression analysis of risk factors for intra-abdominal infection in patients undergoing Hartmann’s procedure between 2007 and 2014

	Univariable analysis			Multivariable analysis		
	Odds ratio	95% CI	*P*	Odds ratio	95% CI	*P*
Age	0.98	0.96–0.99	0.013			
BMI	***1.04***	***1.00–1.08***	***0.048***	1.04	1.00–1.08	0.050
Gender						
Male	1.00			1.00		
Female	***1.59***	***1.10–2.32***	***0.015***	***1.73***	***1.15–2.61***	***0.009***
ASA grade						
1–2	1.00					
3–4	1.14	0.78–1.67	0.492			
T stage (MRI)						
1–3	1.00					
4	***2.026***	***1.33–3.09***	***0.001***	***1.68***	***1.04–2.69***	***0.033***
Distant metastases						
No	1.00					
Yes	1.08	0.65–1.80	0.78			
Distance of tumour from anal verge	0.94	0.88–1.01	0.08			
Preoperative RT						
No	1.00			1.00		
Yes	***2.13***	***1.44–3.15***	***<0.001***	***1.78***	***1.14–2.77***	***0.011***
Rectal wash-out						
No	1.00					
Yes	0.80	0.54–1.17	0.246			
Intra-operative bowel perforation						
No	1.00			1.00		
Yes	***2.17***	***1.26–3.73***	***0.005***	***1.99***	***1.08–3.67***	***0.028***
Local radical resection						
No	1.00					
Yes	0.70	0.41–1.21	0.205			

CI confidence interval, BMI body mass index, ASA American Society of Anesthesiologists, MRI magnetic resonance imaging, RT radiotherapy

**Table 4:** Binary univariable and multivariable logistic regression analysis of risk factors for re-laparotomy in patients undergoing Hartmann’s procedure between 2007 and 2014

	Univariable analysis			Multivariable analysis		
	Odds ratio	95% CI	*P*	Odds ratio	95% CI	*P*
Age	1.00	0.99–1.02	0.701			
BMI	***1.06***	***1.02–1.09***	***0.002***	**1.05**	**1.02–1.09**	**0.003**
Gender						
Male	**1.00**			1.00		
Female	***2.08***	***1.46–2.97***	***<0.001***	**2.06**	**1.41–3.00**	**<0.001**
ASA grade						
1–2	1.00			1.00		
3–4	***1.73***	***1.22–2.46***	***0.002***	***1.62***	***1.12–2.34***	***0.011***
T stage (MRI)						
1–3	1.00					
4	1.11	0.71–1.73	0.642			
Distant metastases						
No	1.00					
Yes	1.22	0.77–1.93	0.394			
Distance of tumour from anal verge	0.99	0.93–1.05	0.666			
Preoperative RT						
No	1.00					
Yes	1.01	0.72–1.42	0.958			
Rectal wash-out						
No	1.00					
Yes	0.76	0.54–1.09	0.134			
Intra-operative bowel perforation						
No	1.00					
Yes	0.94	0.49–1.79	0.841			
Local radical resection						
No	1.00					
Yes	0.82	0.49–1.39	0.459			

CI confidence interval, BMI body mass index, ASA American Society of Anesthesiologists, MRI magnetic resonance imaging, RT radiotherapy

## Discussion and conclusions

In this study of data from a Swedish population-based register collected at the national level, the frequency of postoperative intra-abdominal infection was relatively low in patients treated with HP, despite their age, co-morbidities and advanced disease. The multivariable analysis showed that the risk of intra-abdominal infection increased in women, patients with T4 tumours, those who had intra-operative bowel perforation and those treated with preoperative RT. Patients with a higher ASA score, women and those with an elevated BMI had an increased risk of re-laparotomy. A low HP was not associated with a higher rate of intra-abdominal infection. This study was a large audit of postoperative complications after HP in a national population-based cohort of all patients with rectal cancer. The results strongly indicate that HP is a safe procedure for old and frail patients, and its use is not associated with a high rate of pelvic abscess.

The frequency of intra-abdominal infection (8%) seen in the present study is lower than the pelvic infection rates of 12–33% reported in previous small retrospective cohort series [6–8]. Our results are consistent with those of a recent small population-based study from Sweden [12] and a recent Dutch study [13]. However, some of the risk factors we identified differed from those identified in the Dutch study: the median age in the present study was higher (77 vs. 72 years), and fewer patients had preoperative RT (50% vs. 91%). In addition, stage IV patients were excluded from the Dutch study. That such patients were included in the present study strengthens our conclusion that HP is a relatively safe operation in old and frail patients.

In the multivariable analysis, the risk of intra-abdominal infection increased among those patients undergoing HP who had received preoperative RT (OR 1.78). Similar findings were also reported in the Dutch study, which found that preoperative RT was independently associated with an increased risk of postoperative intra-abdominal abscess that required re-intervention [13]. The Dutch group also recently reported that, in patients with rectal cancer selected for RT, low HP was associated with a lower rate of infectious abdominal complications compared with low anterior resection (AR). In that study, 31% of the patients with rectal cancer were excluded because they had T4 tumours, underwent emergency surgery, did not have RT or had missing values [14]. However, only emergency procedures were excluded in the present study, and the rate of intra-abdominal infection was still low.

One possible reason for the increased risk of intra-abdominal infection after RT is impaired healing of the rectal stump, which is consistent with the increased risk of anastomotic leakage after AR [15]. The size of the staple apparatus and the number of cartridges used may be risk factors for intra-abdominal infection after HP, but these variables were not recorded in the SCRCR. Another possible risk factor for intra-abdominal infection is the level of the rectal transection, with the highest risk after a low HP [6]. In the present study, 62% of the patients underwent a low HP. In the multivariable analysis, we found no association between tumour level and increased risk for intra-abdominal infection indicating that a low HP was not associated with a higher rate of pelvic abscess.

One explanation for the high frequency of intra-operative bowel perforation after HP may be the conversion of an AR to an HP when the surgeon encounters a bowel perforation or other technical difficulty. Another factor may be the surgeon’s level of experience; this is a less likely explanation in our study because 97% of the HP procedures were performed or supervised by a surgeon with colorectal interest or accreditation. However, intra-operative bowel perforation may explain part of the increased risk for developing intra-abdominal infection after HP and may not necessarily be associated with the procedure.

The finding in the multivariable regression analysis that female gender was a risk-factor for intra-abdominal infection and re-laparotomy was surprising. We have not found similar data in the literature on HP and have no explanation for this finding. There were no differences between men and women in terms of age, BMI, ASA grade, metastatic disease or T4 tumours.

Even though the SCRCR has almost complete coverage and the data are recorded prospectively within the registry and continuously updated and validated, our study has some limitations. A study by Gunnarsson et al. of the validation of postoperative complications showed that the validity of the SCRCR is acceptable for severe complications such as intra-abdominal infection, but that the database underestimates the frequency of less serious postoperative morbidities [11]. This is why we chose not to analyse abdominal wound infections in the present study. Data for potential late re-admissions could not be retrieved from the registry, which may have led to an underestimation of the risk of intra-abdominal infection. The mortality increased from 4% at 30 days to 6% at 90 days, and this increase indicates the need for a longer follow-up period for late complications in the registry.

Fifty per cent of the patients in this study had preoperative RT, probably because a relatively high proportion of the patients had metastases. Given the potential oncological benefit of RT, one could speculate that the oncological outcome after HP could be improved by increased use of preoperative RT. However, RT was identified as a risk factor for postoperative intra-abdominal infection. An alternative procedure for patients considered for preoperative RT and HP may be resection of the remaining anorectal stump with an intersphincteric APE. A small Dutch retrospective study of 52 patients compared intersphincteric proctectomy with low HP for low rectal cancer and found no significant difference in the postoperative pelvic abscess rates, which were 17% vs 10%, respectively [16]. The Hartmann’s Procedure or Abdomino-Perineal Excision With Inter-sphincteric Dissection in Rectal Cancer (HAPIrect) trial, (NCT01995396) is an ongoing randomised multi-centre study in Sweden aimed at identifying the optimal surgical treatment method for patients with rectal cancer not suitable for an anastomosis [17].

In conclusion, despite including older patients with advanced cancer and with comorbidities, the frequencies of serious postoperative complications and re-laparotomies were low in patients undergoing HP. Low HP was not associated with an increased rate of intra-abdominal infection, although preoperative RT was identified as a risk factor. An alternative procedure for patients considered for preoperative RT and HP may be resection of the remaining anorectal stump with an intersphincteric APE.
